# Supplementary material for: P-TRAP: a Panicle Trait Phenotyping tool
Source: BMC Plant Biol. 2013 Aug 29;13:122. doi: 10.1186/1471-2229-13-122 (PMC3848748; doi:10.1186/1471-2229-13-122)
Supplement: Additional file 7 — P-TRAP source code. The Netbeans project that contains the source code of the application. [file 1471-2229-13-122-S7.zip › javadoc.zip!/javadoc/org-basmati-workspace/serialized-form.html]

Serialized Form (WorkSpace)


JavaScript is disabled on your browser.


- Package
- Class
- Tree
- Deprecated
- Index
- Help

*org.basmati.workspace 1.0*

- Prev
- Next

- Frames
- No Frames

- All Classes

# Serialized Form

- ## Package org.basmati.workspace

  - ### Class org.basmati.workspace.ColoredImageAction extends org.openide.util.actions.BooleanStateAction implements Serializable

    - ### Serialization Methods

      - #### readExternal

        ```
        public void readExternal(java.io.ObjectInput arg0)
                          throws java.io.IOException,
                                 java.lang.ClassNotFoundException
        ```

        Throws:
        :   `java.io.IOException`
        :   `java.lang.ClassNotFoundException`
      - #### writeExternal

        ```
        public void writeExternal(java.io.ObjectOutput arg0)
                           throws java.io.IOException
        ```

        Throws:
        :   `java.io.IOException`
  - ### Class org.basmati.workspace.EditandViewGrainFileActionDataObject extends org.openide.loaders.MultiDataObject implements Serializable
  - ### Class org.basmati.workspace.GraphEditorTopComponent extends org.openide.windows.TopComponent implements Serializable

    - ### Serialization Methods

      - #### readExternal

        ```
        public void readExternal(java.io.ObjectInput arg0)
                          throws java.io.IOException,
                                 java.lang.ClassNotFoundException
        ```

        Throws:
        :   `java.io.IOException`
        :   `java.lang.ClassNotFoundException`
      - #### writeExternal

        ```
        public void writeExternal(java.io.ObjectOutput arg0)
                           throws java.io.IOException
        ```

        Throws:
        :   `java.io.IOException`
  - ### Class org.basmati.workspace.ImageTypeAction extends org.openide.util.actions.BooleanStateAction implements Serializable

    - ### Serialization Methods

      - #### readExternal

        ```
        public void readExternal(java.io.ObjectInput arg0)
                          throws java.io.IOException,
                                 java.lang.ClassNotFoundException
        ```

        Throws:
        :   `java.io.IOException`
        :   `java.lang.ClassNotFoundException`
      - #### writeExternal

        ```
        public void writeExternal(java.io.ObjectOutput arg0)
                           throws java.io.IOException
        ```

        Throws:
        :   `java.io.IOException`
  - ### Class org.basmati.workspace.ParticlesEditorTopComponent extends org.openide.windows.TopComponent implements Serializable

    - ### Serialization Methods

      - #### readExternal

        ```
        public void readExternal(java.io.ObjectInput arg0)
                          throws java.io.IOException,
                                 java.lang.ClassNotFoundException
        ```

        Throws:
        :   `java.io.IOException`
        :   `java.lang.ClassNotFoundException`
      - #### writeExternal

        ```
        public void writeExternal(java.io.ObjectOutput arg0)
                           throws java.io.IOException
        ```

        Throws:
        :   `java.io.IOException`
  - ### Class org.basmati.workspace.ProcessedRiceImageFileDataObject extends org.openide.loaders.MultiDataObject implements Serializable

    - ### Serialized Fields

      - #### imagefile

        ```
        org.openide.filesystems.FileObject imagefile
        ```
      - #### graphModel

        ```
        org.bioalg.graphprocessor.GraphModel graphModel
        ```
  - ### Class org.basmati.workspace.ResultsEditorTopComponent extends org.openide.windows.TopComponent implements Serializable

    - ### Serialization Methods

      - #### readExternal

        ```
        public void readExternal(java.io.ObjectInput arg0)
                          throws java.io.IOException,
                                 java.lang.ClassNotFoundException
        ```

        Throws:
        :   `java.io.IOException`
        :   `java.lang.ClassNotFoundException`
      - #### writeExternal

        ```
        public void writeExternal(java.io.ObjectOutput arg0)
                           throws java.io.IOException
        ```

        Throws:
        :   `java.io.IOException`
  - ### Class org.basmati.workspace.ScaleSelectorPanel extends javax.swing.JPanel implements Serializable

    - ### Serialized Fields

      - #### jComboBoxUnit

        ```
        javax.swing.JComboBox<E> jComboBoxUnit
        ```
      - #### jLabel1

        ```
        javax.swing.JLabel jLabel1
        ```
      - #### jLabel2

        ```
        javax.swing.JLabel jLabel2
        ```
      - #### jLabel3

        ```
        javax.swing.JLabel jLabel3
        ```
      - #### jLabelAmount

        ```
        javax.swing.JLabel jLabelAmount
        ```
      - #### jLabelPixels

        ```
        javax.swing.JLabel jLabelPixels
        ```
      - #### jTextFieldAmount

        ```
        javax.swing.JTextField jTextFieldAmount
        ```
  - ### Class org.basmati.workspace.ScalingPanel extends javax.swing.JPanel implements Serializable

    - ### Serialized Fields

      - #### jComboBoxScaleRatio

        ```
        javax.swing.JComboBox<E> jComboBoxScaleRatio
        ```
      - #### jLabel1

        ```
        javax.swing.JLabel jLabel1
        ```
  - ### Class org.basmati.workspace.VeiwParticlesAnchors extends org.openide.util.actions.BooleanStateAction implements Serializable

    - ### Serialization Methods

      - #### readExternal

        ```
        public void readExternal(java.io.ObjectInput arg0)
                          throws java.io.IOException,
                                 java.lang.ClassNotFoundException
        ```

        Throws:
        :   `java.io.IOException`
        :   `java.lang.ClassNotFoundException`
      - #### writeExternal

        ```
        public void writeExternal(java.io.ObjectOutput arg0)
                           throws java.io.IOException
        ```

        Throws:
        :   `java.io.IOException`

- Package
- Class
- Tree
- Deprecated
- Index
- Help

*org.basmati.workspace 1.0*

- Prev
- Next

- Frames
- No Frames

- All Classes
